# Relationship between DNA methylation changes and skeletal muscle mass

**DOI:** 10.1186/s12863-023-01152-3

**Published:** 2023-08-31

**Authors:** Jeong-An Gim, Sang-Yeob Lee, Seung Chan Kim, Kyung-Wan Baek, Sung Hyo Seo, Jun-Il Yoo

**Affiliations:** 1 Department of Medical Science Research Center, College of Medicine, Korea University, Seoul, South Korea; 2 Department of Biomedical Research Institute, Gyeongsang National University Hospital, Jinju, South Korea; 3 Department of Theriogenology and Biotechnology, College of Veterinary Medicine, Gyeongsang National University, Jinju, South Korea; 4 Department of Biostatistics Cooperation Center, Gyeongsang National University Hospital, Jinju, South Korea; 5 Department of Physical Education, Gyeongsang National University, Jinju, South Korea; 6 Department of Research Institute of Pharmaceutical Sciences, Gyeongsang National University, Jinju, South Korea; 7 Department of Orthopaedic Surgery, Inha University Hospital, 27 Inhang-ro, Jung-gu, Incheon, 22332 Republic of Korea

**Keywords:** Sarcopenia, DNA methylation, Differentially methylated regions, Muscle mass index, Epigenetics

## Abstract

**Background:**

Sarcopenia is a disease diagnosed in the elderly. In patients with sarcopenia, the muscle mass decreases every year. The occurrence of sarcopenia is greatly affected by extrinsic factors such as eating habits, exercise, and lifestyle. The present study aimed to determine the relationship between muscle mass traits and genes affected by epigenetic factors with three different adjustment methods using Korean Genome and Epidemiology Study (KOGES) data.

**Results:**

We conducted a demographic study and DNA methylation profiling by three studies according to the muscle mass index (MMI) adjustment methods: appendicular skeletal muscle mass divided by body weight (MMI1); appendicular skeletal muscle mass divided by square of height (MMI2); appendicular skeletal muscle mass divided by BMI (MMI3). We analyzed differentially methylated regions (DMRs) for each group. We then restricted our subjects to be top 30% (T30) and bottom 30% (B30) based on each MMI adjustment method. Additionally, we performed enrichment analysis using PathfindR to evaluate the relationship between identified DMRs and sarcopenia. A total of 895 subjects were included in the demographic study. The values of BMI, waist, and hip showed a significant difference in all three groups. Among 446 participants, 44 subjects whose DNA methylation profiles were investigated were included for DNA methylation analysis. The results of enrichment analysis showed differences between groups. In the women group through MMI1 method, only the glutamatergic synapse pathway showed a significant result. In the men group through MMI2 method, the adherens junction pathway was the most significant. Women group through MMI2 method showed similar results, having an enriched Rap1 signaling pathway. In men group through MMI3 method, the Fc epsilon RI signaling pathway was the most enriched. Particularly, the notch signaling pathway was significantly enriched in women group through MMI3 method.

**Conclusion:**

This study presents results about which factor should be concerned first in muscle mass index (MMI) adjustment. The present study suggested that GAB2 and JPH3 in MMI1 method, HLA-DQB1 and TBCD in MMI2 method, GAB2, NDUFB4 and ISPD in MMI3 method are potential genes that can have an impact on muscle mass. It could enable future epigenetic studies of genes based on annotation results. The present study is a nationwide study in Korea with the largest size up to date that compares adjustment indices for MMI in epigenetic research.

**Supplementary Information:**

The online version contains supplementary material available at 10.1186/s12863-023-01152-3.

## Introduction

Although old age is not a sickness, numerous diseases and syndromes are more common in the elderly. Recently, interest in research on sarcopenia is increasing as the number of elderly people increases [1–3]. In Korea, research for establishing the criteria of sarcopenia and their adequacy for diagnosis has been actively underway through policy-making [4]. Sarcopenia has different adjustment indices and diagnostic criteria for each continent according to race, culture, diet, and so on [5]. The Asian Working Group for Sarcopenia (AWGS) consensus has published the diagnostic standard of sarcopenia by height-adjusted muscle mass [6]. However, Korean and Asian studies have reported sarcopenia adjustment indices using weight and body mass index (BMI). Several studies have reported different adjustment methods [7–11]. In addition, it has been reported that the prevalence rate of sarcopenia might vary depending on the diagnosis index [6]. Thus, further research is needed.

Sarcopenia is a disease diagnosed in the elderly. Muscle mass is known to decrease every year since the age of 30. The risk of sarcopenia is rapidly increased during middle age when exercise, hormonal changes, and digestive ability are rapidly decreasing [12–14]. During this period, the risk of metabolic diseases including osteoporosis and hyperlipidemia due to menopause in women is also high [15–17]. In addition, sarcopenia is a disease that is greatly affected by extrinsic factors such as eating habits, exercise, and lifestyle [18–20]. However, studies on the relationship between sarcopenia and extrinsic factors and how it varies depending on the adjustment index in East Asia, including Korea, are insufficient.

Therefore, the present study aimed to determine the relationship between muscle mass traits and epigenetic genes with three different adjustment indices (weight adjustment, square of height adjustment, and BMI adjustment) using the Korean Genome and Epidemiology Study (KOGES) data.

## Materials and methods

### Study subjects

Data used in this study were from the Korean Genome and Epidemiology Study (KoGES) performed by the National Research Institute of Health, Centers for Disease Control and Prevention, Ministry for Health and Welfare, Republic of Korea. The number of baseline participants was 10,030. They lived in Ansan or Ansung located in Gyeonggi Province, South Korea. In this cohort, participants aged 40 to 69 years. 9,351 subjects who had laboratory data were included. Using the bioelectrical impedance analysis (BIA) (body composition analyzer, models ZEUS 9.9, JAWON MEDICAL CO., LTD, Seoul, Korea), skeletal muscle mass was measured. We conducted three studies according to the muscle mass index (MMI) adjustment methods: appendicular skeletal muscle mass divided by body weight (MMI1); appendicular skeletal muscle mass divided by square of height (MMI2); appendicular skeletal muscle mass divided by BMI (MMI3). We then restricted our subjects to be top 30% (T30) and bottom 30% (B30) based on each MMI adjustment method.

The study was approved by the Institutional Review Board (IRB) of Korea University (Approval Number: KUIRB-2020-0191-01). All study subjects provided written informed consent.

### Body composition measurement, demographic factors, and medical history

Method of body composition measurement, demographic factors, and medical history were previously described [21]. All participants attended a community clinic for clinical assessments at each follow-up visit. BMI was calculated as weight in kg divided by the square of height in meters. Weight was determined for an individual wearing light clothes without shoes (barefoot). Waist and hip circumference were also measured. The remaining survey items consisted of drinking & smoking status, level of education, and monthly income. History of hypertension, diabetes, gastritis/stomach ulcer, allergy, myocardial infarction, thyroid disorder, congestive heart failure, coronary artery disease, hyperlipidemia, asthma, chronic lung disorder, peripheral vascular disease, kidney disease, various tumors, cerebrovascular disease, head trauma, urinary tract infection, gout, degenerative arthritis, and rheumatoid arthritis was also taken.

### DNA methylation profiling

The present study collected epidemiology data once every two years. DNA methylation profiles were investigated during the 4th follow-up (2009–2010) for 446 participants. In the DNA methylation study, participants were also restricted to T30 and B30. An Infinium HumanMethylation 450K beadChip (Illumina, Inc., San Diego, CA, USA) was used to obtain KoGES DNA methylation data. Quality control procedure was applied to DNA methylation data. The beta value indicating DNA methylation level was calculated as [ (Methylated reads) / (Unmethylated reads) + (Methylated reads) ]. After filtering, a total of 389,321 CpGs remained for epigenome-wide association analysis. After that, we merged CpG sites data and annotation data of Illumina Human Methylation EPIC manifest package using R. Differentially methylated regions (DMRs) were identified using a t-test performed between T30 group and B30 group using the criteria of *p* < 5*10^-3 and | fold change | > 0.2 to find differentially methylated CpG sites. The gene of annotation data merged with a CpG site with a significant difference in bata value was displayed in a volcano plot. Enrichment analyses of DMRs were performed using the “pathfindR” package which integrates pathway/gene set annotations from sources such as Kyoto Encylopedia of Genes and Genomes (KEGG), Reactome, BioCarta, and Gene Ontology (GO) [22].

### Statistical analysis

Continuous data are reported as mean ± standard deviation and categorical data are reported as n (%). To find any significant difference in baseline characteristics or clinical factors between T30 and B30, unpaired t-test was used for continuous variables if normality assumption was met. Otherwise, Wilcoxon’s rank-sum test was performed. Differences in proportions between T30 and B30 were analyzed using Chi-squared test for categorical variables. If the assumption of Chi-squared test did not meet, Fisher’s exact test was performed.

An unpaired T-test was performed to identify genes from annotation results of methylated CpG sites between muscle mass traits groups. Methylated CpG sites were visualized by a volcano plot. Each CpG site was differentiated by the following criteria: *p* < 5*10^-3 and | fold change | > 0.2.

All statistical analyses were carried out using R software version 4.1.0 (R Core Team. R Foundation for Statistical Computing, Vienna, Austria, 2020). The significance level was set at *p* < 0.05.

## Results

### Demographic characteristics

A total of 9,351 middle-aged people participated in this study. Those in B30 were considered to have insufficient muscle mass, while those in T30 were considered to have sufficient muscle mass. 7,452 people who were not included in T30 or B30 were excluded. A total of 1,004 participants with at least one missing value were also excluded. Finally, a total of 895 subjects (389 males and 506 females) were included in this study. In MMI1 method, there were 215 people with T30 and 174 people with B30 in the male group and 228 people with T30 and 278 people with B30 in the female group. In MMI2 method, there were 169 people with T30 and 220 people with B30 in the male group and 308 people with T30 and 198 people with B30 in the female group. When adjusted for BMI, there were 205 subjects with T30 and 184 subjects with B30 in the male group and 227 people with T30 and 279 people B30 in the female group.

### Demographics according to weight adjustment index (MMI1)

Statistically significant differences in BMI, waist, hip, smoking status, history of gastritis/stomach ulcer, history of hyperlipidemia, history of various tumors, and sedentary life status between T30 group and B30 group were found in men. Particularly, BMI, waist, hip, smoking status, and gastritis/stomach ulcer history showed high significance. Age, BMI, waist, hip, hypertension history, diabetes history, gout history, and degenerative arthritis history showed significant differences between T30 group and B30 group were found in women. Particularly, age, BMI, waist, hip, hypertension history, gout history, and degenerative arthritis history showed high significance (Table 1).

**Table 1:** Demographic study according to weight adjustment index

	Man				Woman			
Characteristic	Q70 over (n = 215)	Q30 under (n = 174)	Total (n = 389)	P-value	Q70 over (n = 228)	Q30 under (n = 278)	Total (n = 506)	P-value
**Age**	52.8 ± 9.3	52.1 ± 9.5	52.5 ± 9.3	0.43	49.3 ± 8.4	54.7 ± 9.0	52.3 ± 9.2	< 0.001***
**BMI**	21.2 ± 2.6	27.5 ± 2.9	24.0 ± 4.1	< 0.001***	21.8 ± 2.5	29.1 ± 3.1	25.8 ± 4.6	< 0.001***
**Waist**	76.6 ± 6.3	90.3 ± 6.1	82.7 ± 9.2	< 0.001***	74.4 ± 7.9	90.0 ± 9.3	83.0 ± 11.6	< 0.001***
**Hip**	89.4 ± 5.6	97.6 ± 5.8	93.1 ± 7.0	< 0.001***	90.2 ± 5.0	99.5 ± 6.2	95.3 ± 7.4	< 0.001***
**Drink**				0.706				0.626
Never	44 (20.5%)	41 (23.6%)	85 (21.9%)		160 (70.2%)	205 (73.7%)	365 (72.1%)	
Former drinker	23 (10.7%)	20 (11.5%)	43 (11.1%)		7 (3.1%)	9 (3.2%)	16 (3.2%)	
Current drinker	148 (68.8%)	113 (64.9%)	261 (67.1%)		61 (26.8%)	64 (23%)	125 (24.7%)	
**Smoke**				< 0.001***				0.209
Never smoked	39 (18.1%)	44 (25.3%)	83 (21.3%)		216 (94.7%)	268 (96.4%)	484 (95.7%)	
Former smokers	56 (26%)	76 (43.7%)	132 (33.9%)		5 (2.2%)	4 (1.4%)	9 (1.8%)	
Current smokers (Sometimes)	10 (4.7%)	5 (2.9%)	15 (3.9%)		1 (0.4%)	4 (1.4%)	5 (1%)	
Current smokers (often)	110 (51.2%)	49 (28.2%)	159 (40.9%)		6 (2.6%)	2 (0.7%)	8 (1.6%)	
**Education level**				0.591				< 0.001***
Under elementary school	49 (22.8%)	41 (23.6%)	90 (23.1%)		73 (32%)	159 (57.2%)	232 (45.8%)	
Middle school	55 (25.6%)	40 (23%)	95 (24.4%)		56 (24.6%)	50 (18%)	106 (20.9%)	
High school	71 (33%)	52 (29.9%)	123 (31.6%)		74 (32.5%)	51 (18.3%)	125 (24.7%)	
Junior college	13 (6%)	4 (2.3%)	17 (4.4%)		8 (3.5%)	2 (0.7%)	10 (2%)	
University	23 (10.7%)	30 (17.2%)	53 (13.6%)		16 (7%)	15 (5.4%)	31 (6.1%)	
Graduate school	4 (1.9%)	7 (4%)	11 (2.8%)		1 (0.4%)	1 (0.4%)	2 (0.4%)	
**Monthly income**				0.958				0.097
< 500 thousand won	34 (15.8%)	26 (14.9%)	60 (15.4%)		39 (17.1%)	79 (28.4%)	118 (23.3%)	
500–1000 thousand won	38 (17.7%)	20 (11.5%)	58 (14.9%)		40 (17.5%)	40 (14.4%)	80 (15.8%)	
1000–1500 thousand won	33 (15.3%)	25 (14.4%)	58 (14.9%)		34 (14.9%)	51 (18.3%)	85 (16.8%)	
1500–2000 thousand won	32 (14.9%)	23 (13.2%)	55 (14.1%)		31 (13.6%)	37 (13.3%)	68 (13.4%)	
2000–3000 thousand won	44 (20.5%)	40 (23%)	84 (21.6%)		39 (17.1%)	45 (16.2%)	84 (16.6%)	
3000–4000 thousand won	21 (9.8%)	25 (14.4%)	46 (11.8%)		22 (9.6%)	18 (6.5%)	40 (7.9%)	
4000–6000 thousand won	10 (4.7%)	9 (5.2%)	19 (4.9%)		18 (7.9%)	7 (2.5%)	25 (4.9%)	
6000 thousand won	3 (1.4%)	6 (3.4%)	9 (2.3%)		5 (2.2%)	1 (0.4%)	6 (1.2%)	
**History of Hypertension**				0.121				< 0.001***
	197 (91.6%)	150 (86.2%)	347 (89.2%)		209 (91.7%)	197 (70.9%)	406 (80.2%)	
Yes	18 (8.4%)	24 (13.8%)	42 (10.8%)		19 (8.3%)	81 (29.1%)	100 (19.8%)	
**History of Diabetes**				0.645				0.015**
No	199 (92.6%)	164 (94.3%)	363 (93.3%)		216 (94.7%)	245 (88.1%)	461 (91.1%)	
Yes	16 (7.4%)	10 (5.7%)	26 (6.7%)		12 (5.3%)	33 (11.9%)	45 (8.9%)	
**History of Gastritis/stomach ulcer**				0.002***				0.059
No	155 (72.1%)	149 (85.6%)	304 (78.1%)		175 (76.8%)	233 (83.8%)	408 (80.6%)	
Yes	60 (27.9%)	25 (14.4%)	85 (21.9%)		53 (23.2%)	45 (16.2%)	98 (19.4%)	
**History of Allergy**				0.789				0.341
No	204 (94.9%)	167 (96%)	371 (95.4%)		210 (92.1%)	263 (94.6%)	473 (93.5%)	
Yes	11 (5.1%)	7 (4%)	18 (4.6%)		18 (7.9%)	15 (5.4%)	33 (6.5%)	
**History of Myocardial infarction**								1
No	214 (99.5%)	173 (99.4%)	387 (99.5%)		226 (99.1%)	276 (99.3%)	502 (99.2%)	
Yes	1 (0.5%)	1 (0.6%)	2 (0.5%)		2 (0.9%)	2 (0.7%)	4 (0.8%)	
**History of Thyroid disease**				0.66				0.357
No	213 (99.1%)	171 (98.3%)	384 (98.7%)		214 (93.9%)	267 (96%)	481 (95.1%)	
Yes	2 (0.9%)	3 (1.7%)	5 (1.3%)		14 (6.1%)	11 (4%)	25 (4.9%)	
**History of Congestive heart failure**				NA				NA
No	215 (100%)	174 (100%)	389 (100%)		228 (100%)	278 (100%)	506 (100%)	
Yes	0 (0%)	0 (0%)	0 (0%)		0 (0%)	0 (0%)	0 (0%)	
**History of Coronary artery disease**				0.256				1
No	212 (98.6%)	174 (100%)	386 (99.2%)		228 (100%)	277 (99.6%)	505 (99.8%)	
Yes	3 (1.4%)	0 (0%)	3 (0.8%)		0 (0%)	1 (0.4%)	1 (0.2%)	
**History of Hyperlipidemia**				0.015*				0.565
No	213 (99.1%)	164 (94.3%)	377 (96.9%)		222 (97.4%)	267 (96%)	489 (96.6%)	
Yes	2 (0.9%)	10 (5.7%)	12 (3.1%)		6 (2.6%)	11 (4%)	17 (3.4%)	
**History of Asthma**				0.521				0.088
No	211 (98.1%)	169 (97.1%)	380 (97.7%)		226 (99.1%)	268 (96.4%)	494 (97.6%)	
Yes	4 (1.9%)	5 (2.9%)	9 (2.3%)		2 (0.9%)	10 (3.6%)	12 (2.4%)	
**History of Chronic lung disease**				1				0.256
No	214 (99.5%)	173 (99.4%)	387 (99.5%)		228 (100%)	275 (98.9%)	503 (99.4%)	
Yes	1 (0.5%)	1 (0.6%)	2 (0.5%)		0 (0%)	3 (1.1%)	3 (0.6%)	
**History of Peripheral vascular disease**				0.447				0.451
No	215 (100%)	173 (99.4%)	388 (99.7%)		227 (99.6%)	278 (100%)	505 (99.8%)	
Yes	0 (0%)	1 (0.6%)	1 (0.3%)		1 (0.4%)	0 (0%)	1 (0.2%)	
**History of Kidney disease**				0.329				0.507
No	214 (99.5%)	171 (98.3%)	385 (99%)		223 (97.8%)	268 (96.4%)	491 (97%)	
Yes	1 (0.5%)	3 (1.7%)	4 (1%)		5 (2.2%)	10 (3.6%)	15 (3%)	
**History of Various tumors**				0.035*				0.219
No	209 (97.2%)	174 (100%)	383 (98.5%)		220 (96.5%)	274 (98.6%)	494 (97.6%)	
Yes	6 (2.8%)	0 (0%)	6 (1.5%)		8 (3.5%)	4 (1.4%)	12 (2.4%)	
**History of Cerebrovascular disease**				0.737				0.23
No	209 (97.2%)	171 (98.3%)	380 (97.7%)		227 (99.6%)	273 (98.2%)	500 (98.8%)	
Yes	6 (2.8%)	3 (1.7%)	9 (2.3%)		1 (0.4%)	5 (1.8%)	6 (1.2%)	
**History of Head trauma**				0.256				NA
No	212 (98.6%)	174 (100%)	386 (99.2%)		228 (100%)	278 (100%)	506 (100%)	
Yes	3 (1.4%)	0 (0%)	3 (0.8%)		0 (0%)	0 (0%)	(0%)	
**History of Urinary tract infection**				0.199				1
No	215 (100%)	172 (98.9%)	387 (99.5%)		226 (99.1%)	276 (99.3%)	502 (99.2%)	
Yes	0 (0%)	2 (1.1%)	2 (0.5%)		2 (0.9%)	2 (0.7%)	4 (0.8%)	
**History of Gout**				0.761				0.003**
No	208 (96.7%)	170 (97.7%)	378 (97.2%)		211 (92.5%)	232 (83.5%)	443 (87.5%)	
Yes	7 (3.3%)	4 (2.3%)	11 (2.8%)		17 (7.5%)	46 (16.5%)	63 (12.5%)	
**History of Degenerative arthritis**				1				< 0.001***
No	206 (95.8%)	167 (96%)	373 (95.9%)		202 (88.6%)	207 (74.5%)	409 (80.8%)	
Yes	9 (4.2%)	7 (4%)	16 (4.1%)		26 (11.4%)	71 (25.5%)	97 (19.2%)	
**History of Rheumatoid arthritis**				1				0.737
No	213 (99.1%)	173 (99.4%)	386 (99.2%)		208 (91.2%)	257 (92.4%)	465 (91.9%)	
Yes	2 (0.9%)	1 (0.6%)	3 (0.8%)		20 (8.8%)	21 (7.6%)	41 (8.1%)	
**Steady state**				0.994				0.988
Never	65 (30.2%)	52 (29.9%)	117 (30.1%)		64 (28.1%)	68 (24.5%)	132 (26.1%)	
< 30′	26 (12.1%)	20 (11.5%)	46 (11.8%)		43 (18.9%)	44 (15.8%)	87 (17.2%)	
30′~60′	46 (21.4%)	33 (19%)	79 (20.3%)		38 (16.7%)	52 (18.7%)	90 (17.8%)	
60′~90′	17 (7.9%)	22 (12.6%)	39 (10%)		27 (11.8%)	30 (10.8%)	57 (11.3%)	
90′~120′	22 (10.2%)	11 (6.3%)	33 (8.5%)		14 (6.1%)	20 (7.2%)	34 (6.7%)	
120′~180′	16 (7.4%)	17 (9.8%)	33 (8.5%)		23 (10.1%)	26 (9.4%)	49 (9.7%)	
180′~240′	11 (5.1%)	9 (5.2%)	20 (5.1%)		9 (3.9%)	17 (6.1%)	26 (5.1%)	
240′~300′	5 (2.3%)	4 (2.3%)	9 (2.3%)		2 (0.9%)	8 (2.9%)	10 (2%)	
> 300′	7 (3.3%)	6 (3.4%)	13 (3.3%)		8 (3.5%)	13 (4.7%)	21 (4.2%)	
**Sedentary life**				0.028*				0.257
Never	18 (8.4%)	8 (4.6%)	26 (6.7%)		15 (6.6%)	11 (4%)	26 (5.1%)	
< 30′	13 (6%)	10 (5.7%)	23 (5.9%)		10 (4.4%)	20 (7.2%)	30 (5.9%)	
30′~60′	16 (7.4%)	7 (4%)	23 (5.9%)		20 (8.8%)	32 (11.5%)	52 (10.3%)	
60′~90′	25 (11.6%)	9 (5.2%)	34 (8.7%)		23 (10.1%)	32 (11.5%)	55 (10.9%)	
90′~120′	19 (8.8%)	12 (6.9%)	31 (8%)		25 (11%)	18 (6.5%)	43 (8.5%)	
120′~180′	34 (15.8%)	24 (13.8%)	58 (14.9%)		35 (15.4%)	33 (11.9%)	68 (13.4%)	
180′~240′	23 (10.7%)	23 (13.2%)	46 (11.8%)		31 (13.6%)	47 (16.9%)	78 (15.4%)	
240′~300′	12 (5.6%)	9 (5.2%)	21 (5.4%)		21 (9.2%)	21 (7.6%)	42 (8.3%)	
> 300′	55 (25.6%)	72 (41.4%)	127 (32.6%)		48 (21.1%)	64 (23%)	112 (22.1%)	
**Mild exercise**				0.522				0.915
Never	25 (11.6%)	17 (9.8%)	42 (10.8%)		5 (2.2%)	3 (1.1%)	8 (1.6%)	
< 30′	25 (11.6%)	25 (14.4%)	50 (12.9%)		11 (4.8%)	20 (7.2%)	31 (6.1%)	
30′~60′	31 (14.4%)	23 (13.2%)	54 (13.9%)		31 (13.6%)	49 (17.6%)	80 (15.8%)	
60′~90′	19 (8.8%)	24 (13.8%)	43 (11.1%)		35 (15.4%)	52 (18.7%)	87 (17.2%)	
90′~120′	31 (14.4%)	22 (12.6%)	53 (13.6%)		29 (12.7%)	29 (10.4%)	58 (11.5%)	
120′~180′	19 (8.8%)	16 (9.2%)	35 (9%)		36 (15.8%)	30 (10.8%)	66 (13%)	
180′~240′	15 (7%)	18 (10.3%)	33 (8.5%)		27 (11.8%)	37 (13.3%)	64 (12.6%)	
240′~300′	10 (4.7%)	4 (2.3%)	14 (3.6%)		10 (4.4%)	16 (5.8%)	26 (5.1%)	
> 300′	40 (18.6%)	25 (14.4%)	65 (16.7%)		44 (19.3%)	42 (15.1%)	86 (17%)	
**Moderate exercise**				0.927				0.951
Never	94 (43.7%)	86 (49.4%)	180 (46.3%)		101 (44.3%)	149 (53.6%)	250 (49.4%)	
< 30′	22 (10.2%)	23 (13.2%)	45 (11.6%)		40 (17.5%)	34 (12.2%)	74 (14.6%)	
30′~60′	22 (10.2%)	20 (11.5%)	42 (10.8%)		30 (13.2%)	38 (13.7%)	68 (13.4%)	
60′~90′	19 (8.8%)	16 (9.2%)	35 (9%)		23 (10.1%)	29 (10.4%)	52 (10.3%)	
90′~120′	15 (7%)	11 (6.3%)	26 (6.7%)		10 (4.4%)	8 (2.9%)	18 (3.6%)	
120′~180′	12 (5.6%)	7 (4%)	19 (4.9%)		10 (4.4%)	9 (3.2%)	19 (3.8%)	
180′~240′	8 (3.7%)	4 (2.3%)	12 (3.1%)		3 (1.3%)	4 (1.4%)	7 (1.4%)	
240′~300′	7 (3.3%)	3 (1.7%)	10 (2.6%)		3 (1.3%)	2 (0.7%)	5 (1%)	
> 300′	16 (7.4%)	4 (2.3%)	20 (5.1%)		8 (3.5%)	5 (1.8%)	13 (2.6%)	
**Intense exercise**				0.006**				0.822
Never	92 (42.8%)	119 (68.4%)	211 (54.2%)		148 (64.9%)	195 (70.1%)	343 (67.8%)	
< 30′	13 (6%)	7 (4%)	20 (5.1%)		8 (3.5%)	10 (3.6%)	18 (3.6%)	
30′~60′	8 (3.7%)	3 (1.7%)	11 (2.8%)		8 (3.5%)	9 (3.2%)	17 (3.4%)	
60′~90′	6 (2.8%)	4 (2.3%)	10 (2.6%)		11 (4.8%)	10 (3.6%)	21 (4.2%)	
90′~120′	13 (6%)	2 (1.1%)	15 (3.9%)		5 (2.2%)	3 (1.1%)	8 (1.6%)	
120′~180′	11 (5.1%)	8 (4.6%)	19 (4.9%)		6 (2.6%)	8 (2.9%)	14 (2.8%)	
180′~240′	6 (2.8%)	6 (3.4%)	12 (3.1%)		3 (1.3%)	4 (1.4%)	7 (1.4%)	
240′~300′	11 (5.1%)	2 (1.1%)	13 (3.3%)		4 (1.8%)	8 (2.9%)	12 (2.4%)	
> 300′	55 (25.6%)	23 (13.2%)	78 (20.1%)		35 (15.4%)	31 (11.2%)	66 (13%)	

*p < 0.05, **p < 0.01, ***p < 0.001

### Demographics according to the square of height adjustment index (MMI2)

Statistically significant differences in age, BMI, waist, hip, history of hyperlipidemia, and sedentary life status between T30 group and B30 group were found in men. Particularly, age, BMI, waist, and hip showed high significance with *p*-value < 0.005. Age, BMI, waist, hip, and hypertension history showed significant differences T30 group and B30 group in women (Table 2).

**Table 2:** Demographic study according to square of height adjustment index

	Man				Woman				
Characteristic	Q70 over (n = 169)	Q30 under (n = 220)	Total (n = 389)	P-value	Q70 over (n = 308)	Q30 under (n = 198)	Total (n = 506)	P-value	
**Age**	48.5 ± 7.5	55.5 ± 9.5	52.5 ± 9.3	< 0.001***	52.1 ± 8.8	52.5 ± 9.6	52.3 ± 9.2	0.957	
**BMI**	27.9 ± 2.6	21.1 ± 2.3	24.0 ± 4.1	< 0.001***	28.7 ± 3.3	21.4 ± 2.5	25.8 ± 4.6	< 0.001***	
**Waist**	90.1 ± 6.5	77.1 ± 6.7	82.7 ± 9.2	< 0.001***	88.7 ± 9.6	74.1 ± 8.6	83.0 ± 11.6	< 0.001***	
**Hip**	98.6 ± 5.0	88.8 ± 5.0	93.1 ± 7.0	< 0.001***	99.0 ± 6.3	89.6 ± 4.8	95.3 ± 7.4	< 0.001***	
**Drink**				0.312				0.97	
Never	38 (22.5%)	47 (21.4%)	85 (21.9%)		221 (71.8%)	144 (72.7%)	365 (72.1%)		
Former drinker	14 (8.3%)	29 (13.2%)	43 (11.1%)		10 (3.2%)	6 (3%)	16 (3.2%)		
Current drinker	117 (69.2%)	144 (65.5%)	261 (67.1%)		77 (25%)	48 (24.2%)	125 (24.7%)		
**Smoke**				0.253				0.204	
Never smoked	39 (23.1%)	44 (20%)	83 (21.3%)		297 (96.4%)	187 (94.4%)	484 (95.7%)		
Former smokers	64 (37.9%)	68 (30.9%)	132 (33.9%)		6 (1.9%)	3 (1.5%)	9 (1.8%)		
Current smokers (Sometimes)	5 (3%)	10 (4.5%)	15 (3.9%)		3 (1%)	2 (1%)	5 (1%)		
Current smokers (often)	61 (36.1%)	98 (44.5%)	159 (40.9%)		2 (0.6%)	6 (3%)	8 (1.6%)		
**Education level**				0.002**				0.121	
Under elementary school	23 (13.6%)	67 (30.5%)	90 (23.1%)		152 (49.4%)	80 (40.4%)	232 (45.8%)		
Middle school	37 (21.9%)	58 (26.4%)	95 (24.4%)		63 (20.5%)	43 (21.7%)	106 (20.9%)		
High school	64 (37.9%)	59 (26.8%)	123 (31.6%)		72 (23.4%)	53 (26.8%)	125 (24.7%)		
Junior college	9 (5.3%)	8 (3.6%)	17 (4.4%)		6 (1.9%)	4 (2%)	10 (2%)		
University	26 (15.4%)	27 (12.3%)	53 (13.6%)		13 (4.2%)	18 (9.1%)	31 (6.1%)		
Graduate school	10 (5.9%)	1 (0.5%)	11 (2.8%)		2 (0.6%)	0 (0%)	2 (0.4%)		
**Monthly income**				< 0.001***				0.651	
< 500 thousand won	13 (7.7%)	47 (21.4%)	60 (15.4%)		73 (23.7%)	45 (22.7%)	118 (23.3%)		
500–1000 thousand won	15 (8.9%)	43 (19.5%)	58 (14.9%)		47 (15.3%)	33 (16.7%)	80 (15.8%)		
1000–1500 thousand won	28 (16.6%)	30 (13.6%)	58 (14.9%)		56 (18.2%)	29 (14.6%)	85 (16.8%)		
1500–2000 thousand won	28 (16.6%)	27 (12.3%)	55 (14.1%)		48 (15.6%)	20 (10.1%)	68 (13.4%)		
2000–3000 thousand won	38 (22.5%)	46 (20.9%)	84 (21.6%)		50 (16.2%)	34 (17.2%)	84 (16.6%)		
3000–4000 thousand won	27 (16%)	19 (8.6%)	46 (11.8%)		20 (6.5%)	20 (10.1%)	40 (7.9%)		
4000–6000 thousand won	12 (7.1%)	7 (3.2%)	19 (4.9%)		13 (4.2%)	12 (6.1%)	25 (4.9%)		
6000 thousand won	8 (4.7%)	1 (0.5%)	9 (2.3%)		1 (0.3%)	5 (2.5%)	6 (1.2%)		
**History of Hypertension**				0.161				0.004**	
No	146 (86.4%)	201 (91.4%)	347 (89.2%)		234 (76%)	172 (86.9%)	406 (80.2%)		
Yes	23 (13.6%)	19 (8.6%)	42 (10.8%)		74 (24%)	26 (13.1%)	100 (19.8%)		
**History of Diabetes**				0.933				0.189	
No	157 (92.9%)	206 (93.6%)	363 (93.3%)		276 (89.6%)	185 (93.4%)	461 (91.1%)		
Yes	12 (7.1%)	14 (6.4%)	26 (6.7%)		32 (10.4%)	13 (6.6%)	45 (8.9%)		
**History of Gastritis/stomach ulcer**				0.396				0.235	
No	136 (80.5%)	168 (76.4%)	304 (78.1%)		254 (82.5%)	154 (77.8%)	408 (80.6%)		
Yes	33 (19.5%)	52 (23.6%)	85 (21.9%)		54 (17.5%)	44 (22.2%)	98 (19.4%)		
**History of Allergy**				0.876				0.879	
No	162 (95.9%)	209 (95%)	371 (95.4%)		287 (93.2%)	186 (93.9%)	473 (93.5%)		
Yes	7 (4.1%)	11 (5%)	18 (4.6%)		21 (6.8%)	12 (6.1%)	33 (6.5%)		
**History of Myocardial infarction**				1				0.159	
No	168 (99.4%)	219 (99.5%)	387 (99.5%)		304 (98.7%)	198 (100%)	502 (99.2%)		
Yes	1 (0.6%)	1 (0.5%)	2 (0.5%)		4 (1.3%)	0 (0%)	4 (0.8%)		
**History of Thyroid disease**				1				0.47	
No	167 (98.8%)	217 (98.6%)	384 (98.7%)		295 (95.8%)	186 (93.9%)	481 (95.1%)		
Yes	2 (1.2%)	3 (1.4%)	5 (1.3%)		13 (4.2%)	12 (6.1%)	25 (4.9%)		
**History of Congestive heart failure**				NA				NA	
No	169 (100%)	220 (100%)	389 (100%)		308 (100%)	198 (100%)	506 (100%)		
Yes	0 (0%)	0 (0%)	0 (0%)		0 (0%)	0 (0%)	0 (0%)		
**History of Coronary artery disease**				1				1	
No	168 (99.4%)	218 (99.1%)	386 (99.2%)		307 (99.7%)	198 (100%)	505 (99.8%)		
Yes	1 (0.6%)	2 (0.9%)	3 (0.8%)		1 (0.3%)	0 (0%)	1 (0.2%)		
**History of Hyperlipidemia**				0.011**				1	
No	159 (94.1%)	218 (99.1%)	377 (96.9%)		298 (96.8%)	191 (96.5%)	489 (96.6%)		
Yes	10 (5.9%)	2 (0.9%)	12 (3.1%)		10 (3.2%)	7 (3.5%)	17 (3.4%)		
**History of Asthma**				0.084				0.139	
No	168 (99.4%)	212 (96.4%)	380 (97.7%)		298 (96.8%)	196 (99%)	494 (97.6%)		
Yes	1 (0.6%)	8 (3.6%)	9 (2.3%)		10 (3.2%)	2 (1%)	12 (2.4%)		
**History of Chronic lung disease**				0.507				0.564	
No	169 (100%)	218 (99.1%)	387 (99.5%)		307 (99.7%)	196 (99%)	503 (99.4%)		
Yes	0 (0%)	2 (0.9%)	2 (0.5%)		1 (0.3%)	2 (1%)	3 (0.6%)		
**History of Peripheral vascular disease**				1				0.391	
No	169 (100%)	219 (99.5%)	388 (99.7%)		308 (100%)	197 (99.5%)	505 (99.8%)		
Yes	0 (0%)	1 (0.5%)	1 (0.3%)		0 (0%)	1 (0.5%)	1 (0.2%)		
**History of Kidney disease**				1				0.203	
No	167 (98.8%)	218 (99.1%)	385 (99%)		296 (96.1%)	195 (98.5%)	491 (97%)		
Yes	2 (1.2%)	2 (0.9%)	4 (1%)		12 (3.9%)	3 (1.5%)	15 (3%)		
**History of Various tumors**				0.239				0.552	
No	168 (99.4%)	215 (97.7%)	383 (98.5%)		302 (98.1%)	192 (97%)	494 (97.6%)		
Yes	1 (0.6%)	5 (2.3%)	6 (1.5%)		6 (1.9%)	6 (3%)	12 (2.4%)		
**History of Cerebrovascular disease**				0.31				1	
No	167 (98.8%)	213 (96.8%)	380 (97.7%)		304 (98.7%)	196 (99%)	500 (98.8%)		
Yes	2 (1.2%)	7 (3.2%)	9 (2.3%)		4 (1.3%)	2 (1%)	6 (1.2%)		
**History of Head trauma**				1					
No	168 (99.4%)	218 (99.1%)	386 (99.2%)		308 (100%)	198 (100%)	506 (100%)		
Yes	1 (0.6%)	2 (0.9%)	3 (0.8%)						
**History of Urinary tract infection**				1				0.305	
No	168 (99.4%)	219 (99.5%)	387 (99.5%)		307 (99.7%)	195 (98.5%)	502 (99.2%)		
Yes	1 (0.6%)	1 (0.5%)	2 (0.5%)		1 (0.3%)	3 (1.5%)	4 (0.8%)		
**History of Gout**				0.763				0.967	
No	165 (97.6%)	213 (96.8%)	378 (97.2%)		269 (87.3%)	174 (87.9%)	443 (87.5%)		
Yes	4 (2.4%)	7 (3.2%)	11 (2.8%)		39 (12.7%)	24 (12.1%)	63 (12.5%)		
**History of Degenerative arthritis**				0.777				0.029	
No	161 (95.3%)	212 (96.4%)	373 (95.9%)		239 (77.6%)	170 (85.9%)	409 (80.8%)		
Yes	8 (4.7%)	8 (3.6%)	16 (4.1%)		69 (22.4%)	28 (14.1%)	97 (19.2%)		
**History of Rheumatoid arthritis**				1				0.137	
No	168 (99.4%)	218 (99.1%)	386 (99.2%)		288 (93.5%)	177 (89.4%)	465 (91.9%)		
Yes	1 (0.6%)	2 (0.9%)	3 (0.8%)		20 (6.5%)	21 (10.6%)	41 (8.1%)		
**Steady state**				0.964				0.998	
Never	46 (27.2%)	71 (32.3%)	117 (30.1%)		79 (25.6%)	53 (26.8%)	132 (26.1%)		
< 30′	27 (16%)	19 (8.6%)	46 (11.8%)		52 (16.9%)	35 (17.7%)	87 (17.2%)		
30′~60′	36 (21.3%)	43 (19.5%)	79 (20.3%)		59 (19.2%)	31 (15.7%)	90 (17.8%)		
60′~90′	17 (10.1%)	22 (10%)	39 (10%)		33 (10.7%)	24 (12.1%)	57 (11.3%)		
90′~120′	11 (6.5%)	22 (10%)	33 (8.5%)		23 (7.5%)	11 (5.6%)	34 (6.7%)		
120′~180′	14 (8.3%)	19 (8.6%)	33 (8.5%)		25 (8.1%)	24 (12.1%)	49 (9.7%)		
180′~240′	10 (5.9%)	10 (4.5%)	20 (5.1%)		18 (5.8%)	8 (4%)	26 (5.1%)		
240′~300′	3 (1.8%)	6 (2.7%)	9 (2.3%)		7 (2.3%)	3 (1.5%)	10 (2%)		
> 300′	5 (3%)	8 (3.6%)	13 (3.3%)		12 (3.9%)	9 (4.5%)	21 (4.2%)		
**Sedentary life**				0.028*				0.287	
Never	6 (3.6%)	20 (9.1%)	26 (6.7%)		11 (3.6%)	15 (7.6%)	26 (5.1%)		
< 30′	10 (5.9%)	13 (5.9%)	23 (5.9%)		21 (6.8%)	9 (4.5%)	30 (5.9%)		
30′~60′	6 (3.6%)	17 (7.7%)	23 (5.9%)		34 (11%)	18 (9.1%)	52 (10.3%)		
60′~90′	15 (8.9%)	19 (8.6%)	34 (8.7%)		34 (11%)	21 (10.6%)	55 (10.9%)		
90′~120′	10 (5.9%)	21 (9.5%)	31 (8%)		20 (6.5%)	23 (11.6%)	43 (8.5%)		
120′~180′	23 (13.6%)	35 (15.9%)	58 (14.9%)		42 (13.6%)	26 (13.1%)	68 (13.4%)		
180′~240′	19 (11.2%)	27 (12.3%)	46 (11.8%)		50 (16.2%)	28 (14.1%)	78 (15.4%)		
240′~300′	9 (5.3%)	12 (5.5%)	21 (5.4%)		25 (8.1%)	17 (8.6%)	42 (8.3%)		
> 300′	71 (42%)	56 (25.5%)	127 (32.6%)		71 (23.1%)	41 (20.7%)	112 (22.1%)		
**Mild exercise**				0.595				0.998	
Never	17 (10.1%)	25 (11.4%)	42 (10.8%)		4 (1.3%)	4 (2%)	8 (1.6%)		
< 30′	28 (16.6%)	22 (10%)	50 (12.9%)		20 (6.5%)	11 (5.6%)	31 (6.1%)		
30′~60′	27 (16%)	27 (12.3%)	54 (13.9%)		50 (16.2%)	30 (15.2%)	80 (15.8%)		
60′~90′	17 (10.1%)	26 (11.8%)	43 (11.1%)		48 (15.6%)	39 (19.7%)	87 (17.2%)		
90′~120′	22 (13%)	31 (14.1%)	53 (13.6%)		36 (11.7%)	22 (11.1%)	58 (11.5%)		
120′~180′	15 (8.9%)	20 (9.1%)	35 (9%)		38 (12.3%)	28 (14.1%)	66 (13%)		
180′~240′	11 (6.5%)	22 (10%)	33 (8.5%)		44 (14.3%)	20 (10.1%)	64 (12.6%)		
240′~300′	5 (3%)	9 (4.1%)	14 (3.6%)		17 (5.5%)	9 (4.5%)	26 (5.1%)		
> 300′	27 (16%)	38 (17.3%)	65 (16.7%)		51 (16.6%)	35 (17.7%)	86 (17%)		
**Moderate exercise**				0.984				0.979	
Never	76 (45%)	104 (47.3%)	180 (46.3%)		156 (50.6%)	94 (47.5%)	250 (49.4%)		
< 30′	21 (12.4%)	24 (10.9%)	45 (11.6%)		40 (13%)	34 (17.2%)	74 (14.6%)		
30′~60′	22 (13%)	20 (9.1%)	42 (10.8%)		45 (14.6%)	23 (11.6%)	68 (13.4%)		
60′~90′	17 (10.1%)	18 (8.2%)	35 (9%)		35 (11.4%)	17 (8.6%)	52 (10.3%)		
90′~120′	10 (5.9%)	16 (7.3%)	26 (6.7%)		10 (3.2%)	8 (4%)	18 (3.6%)		
120′~180′	10 (5.9%)	9 (4.1%)	19 (4.9%)		8 (2.6%)	11 (5.6%)	19 (3.8%)		
180′~240′	4 (2.4%)	8 (3.6%)	12 (3.1%)		4 (1.3%)	3 (1.5%)	7 (1.4%)		
240′~300′	4 (2.4%)	6 (2.7%)	10 (2.6%)		3 (1%)	2 (1%)	5 (1%)		
> 300′	5 (3%)	15 (6.8%)	20 (5.1%)		7 (2.3%)	6 (3%)	13 (2.6%)		
**Intense exercise**				0.295				0.723	
Never	105 (62.1%)	106 (48.2%)	211 (54.2%)		200 (64.9%)	143 (72.2%)	343 (67.8%)		
< 30′	10 (5.9%)	10 (4.5%)	20 (5.1%)		13 (4.2%)	5 (2.5%)	18 (3.6%)		
30′~60′	6 (3.6%)	5 (2.3%)	11 (2.8%)		13 (4.2%)	4 (2%)	17 (3.4%)		
60′~90′	7 (4.1%)	3 (1.4%)	10 (2.6%)		13 (4.2%)	8 (4%)	21 (4.2%)		
90′~120′	4 (2.4%)	11 (5%)	15 (3.9%)		5 (1.6%)	3 (1.5%)	8 (1.6%)		
120′~180′	6 (3.6%)	13 (5.9%)	19 (4.9%)		9 (2.9%)	5 (2.5%)	14 (2.8%)		
180′~240′	5 (3%)	7 (3.2%)	12 (3.1%)		6 (1.9%)	1 (0.5%)	7 (1.4%)		
240′~300′	3 (1.8%)	10 (4.5%)	13 (3.3%)		8 (2.6%)	4 (2%)	12 (2.4%)		
> 300′	23 (13.6%)	55 (25%)	78 (20.1%)		41 (13.3%)	25 (12.6%)	66 (13%)		

### Demographics according to BMI adjustment index (MMI3)

Statistically significant differences in BMI, waist, hip, smoking status, history of gastritis/stomach ulcer, and history of hyperlipidemia between T30 group and B30 group were found for men. Particularly, BMI, waist, hip, smoking status, and history of gastritis/stomach ulcer showed high significance with p-values less than 0.005. Age, BMI, waist, hip, history of hypertension, history of diabetes, history of gout, and history of degenerative arthritis were significantly different between T30 group and B30 group in women. Particularly, age, BMI, waist, hip, hypertension history, gout history, and degenerative arthritis history showed high significance (Table 3).

**Table 3:** Demographic study according to BMI adjustment index

	Man				Woman				
Characteristic	Q70 over (n = 205)	Q30 under (n = 184)	Total (n = 389)	P-value	Q70 over (n = 227)	Q30 under (n = 279)	Total (n = 506)	P-value	
**Age**	52.0 ± 9.0	53.0 ± 9.7	52.5 ± 9.3	0.367	49.2 ± 8.4	54.7 ± 9.0	52.3 ± 9.2	< 0.001***	
**BMI**	21.4 ± 2.7	26.9 ± 3.5	24.0 ± 4.1	< 0.001***	21.9 ± 2.7	29.0 ± 3.2	25.8 ± 4.6	< 0.001***	
**Waist**	77.1 ± 6.4	89.1 ± 7.7	82.7 ± 9.2	< 0.001***	74.7 ± 8.2	89.7 ± 9.5	83.0 ± 11.6	< 0.001***	
**Hip**	90.1 ± 5.7	96.4 ± 6.8	93.1 ± 7.0	< 0.001***	90.6 ± 5.4	99.2 ± 6.5	95.3 ± 7.4	< 0.001***	
**Drink**				0.323				0.719	
Never	42 (20.5%)	43 (23.4%)	85 (21.9%)		160 (70.5%)	205 (73.5%)	365 (72.1%)		
Former drinker	19 (9.3%)	24 (13%)	43 (11.1%)		7 (3.1%)	9 (3.2%)	16 (3.2%)		
Current drinker	144 (70.2%)	117 (63.6%)	261 (67.1%)		60 (26.4%)	65 (23.3%)	125 (24.7%)		
**Smoke**				< 0.001***				0.209	
Never smoked	38 (18.5%)	45 (24.5%)	83 (21.3%)		215 (94.7%)	269 (96.4%)	484 (95.7%)		
Former smokers	54 (26.3%)	78 (42.4%)	132 (33.9%)		5 (2.2%)	4 (1.4%)	9 (1.8%)		
Current smokers (Sometimes)	8 (3.9%)	7 (3.8%)	15 (3.9%)		1 (0.4%)	4 (1.4%)	5 (1%)		
Current smokers (often)	105 (51.2%)	54 (29.3%)	159 (40.9%)		6 (2.6%)	2 (0.7%)	8 (1.6%)		
**Education level**				0.237				0.002**	
Under elementary school	42 (20.5%)	48 (26.1%)	90 (23.1%)		74 (32.6%)	158 (56.6%)	232 (45.8%)		
Middle school	54 (26.3%)	41 (22.3%)	95 (24.4%)		54 (23.8%)	52 (18.6%)	106 (20.9%)		
High school	69 (33.7%)	54 (29.3%)	123 (31.6%)		74 (32.6%)	51 (18.3%)	125 (24.7%)		
Junior college	12 (5.9%)	5 (2.7%)	17 (4.4%)		8 (3.5%)	2 (0.7%)	10 (2%)		
University	23 (11.2%)	30 (16.3%)	53 (13.6%)		16 (7%)	15 (5.4%)	31 (6.1%)		
Graduate school	5 (2.4%)	6 (3.3%)	11 (2.8%)		1 (0.4%)	1 (0.4%)	2 (0.4%)		
**Monthly income**				0.882				0.168	
< 500 thousand won	30 (14.6%)	30 (16.3%)	60 (15.4%)		39 (17.2%)	79 (28.3%)	118 (23.3%)		
500–1000 thousand won	35 (17.1%)	23 (12.5%)	58 (14.9%)		38 (16.7%)	42 (15.1%)	80 (15.8%)		
1000–1500 thousand won	31 (15.1%)	27 (14.7%)	58 (14.9%)		33 (14.5%)	52 (18.6%)	85 (16.8%)		
1500–2000 thousand won	31 (15.1%)	24 (13%)	55 (14.1%)		34 (15%)	34 (12.2%)	68 (13.4%)		
2000–3000 thousand won	42 (20.5%)	42 (22.8%)	84 (21.6%)		40 (17.6%)	44 (15.8%)	84 (16.6%)		
3000–4000 thousand won	21 (10.2%)	25 (13.6%)	46 (11.8%)		21 (9.3%)	19 (6.8%)	40 (7.9%)		
4000–6000 thousand won	10 (4.9%)	9 (4.9%)	19 (4.9%)		17 (7.5%)	8 (2.9%)	25 (4.9%)		
6000 thousand won	5 (2.4%)	4 (2.2%)	9 (2.3%)		5 (2.2%)	1 (0.4%)	6 (1.2%)		
**History of Hypertension**				0.129				< 0.001***	
No	188 (91.7%)	159 (86.4%)	347 (89.2%)		210 (92.5%)	196 (70.3%)	406 (80.2%)		
Yes	17 (8.3%)	25 (13.6%)	42 (10.8%)		17 (7.5%)	83 (29.7%)	100 (19.8%)		
**History of Diabetes**				0.746				0.036*	
No	190 (92.7%)	173 (94%)	363 (93.3%)		214 (94.3%)	247 (88.5%)	461 (91.1%)		
Yes	15 (7.3%)	11 (6%)	26 (6.7%)		13 (5.7%)	32 (11.5%)	45 (8.9%)		
**History of Gastritis/stomach ulcer**				0.001***				0.054	
No	146 (71.2%)	158 (85.9%)	304 (78.1%)		174 (76.7%)	234 (83.9%)	408 (80.6%)		
Yes	59 (28.8%)	26 (14.1%)	85 (21.9%)		53 (23.3%)	45 (16.1%)	98 (19.4%)		
**History of Allergy**				0.995				0.329	
No	195 (95.1%)	176 (95.7%)	371 (95.4%)		209 (92.1%)	264 (94.6%)	473 (93.5%)		
Yes	10 (4.9%)	8 (4.3%)	18 (4.6%)		18 (7.9%)	15 (5.4%)	33 (6.5%)		
**History of Myocardial infarction**				1				1	
No	204 (99.5%)	183 (99.5%)	387 (99.5%)		225 (99.1%)	277 (99.3%)	502 (99.2%)		
Yes	1 (0.5%)	1 (0.5%)	2 (0.5%)		2 (0.9%)	2 (0.7%)	4 (0.8%)		
**History of Thyroid disease**				0.671				0.346	
No	203 (99%)	181 (98.4%)	384 (98.7%)		213 (93.8%)	268 (96.1%)	481 (95.1%)		
Yes	2 (1%)	3 (1.6%)	5 (1.3%)		14 (6.2%)	11 (3.9%)	25 (4.9%)		
**History of Congestive heart failure**				NA				NA	
No	205 (100%)	184 (100%)	389 (100%)		227 (100%)	279 (100%)	506 (100%)		
Yes	0 (0%)	0 (0%)	0 (0%)		0 (0%)	0 (0%)	0 (0%)		
**History of Coronary artery disease**				1				1	
No	203 (99%)	183 (99.5%)	386 (99.2%)		227 (100%)	278 (99.6%)	505 (99.8%)		
Yes	2 (1%)	1 (0.5%)	3 (0.8%)		0 (0%)	1 (0.4%)	1 (0.2%)		
**History of Hyperlipidemia**				0.025*				0.576	
No	203 (99%)	174 (94.6%)	377 (96.9%)		221 (97.4%)	268 (96.1%)	489 (96.6%)		
Yes	2 (1%)	10 (5.4%)	12 (3.1%)		6 (2.6%)	11 (3.9%)	17 (3.4%)		
**History of Asthma**				0.091				0.09	
No	203 (99%)	177 (96.2%)	380 (97.7%)		225 (99.1%)	269 (96.4%)	494 (97.6%)		
Yes	2 (1%)	7 (3.8%)	9 (2.3%)		2 (0.9%)	10 (3.6%)	12 (2.4%)		
**History of Chronic lung disease**				1				0.256	
No	204 (99.5%)	183 (99.5%)	387 (99.5%)		227 (100%)	276 (98.9%)	503 (99.4%)		
Yes	1 (0.5%)	1 (0.5%)	2 (0.5%)		0 (0%)	3 (1.1%)	3 (0.6%)		
**History of Peripheral vascular disease**				0.473				0.449	
No	205 (100%)	183 (99.5%)	388 (99.7%)		226 (99.6%)	279 (100%)	505 (99.8%)		
Yes	0 (0%)	1 (0.5%)	1 (0.3%)		1 (0.4%)	0 (0%)	1 (0.2%)		
**History of Kidney disease**				0.348				0.517	
No	204 (99.5%)	181 (98.4%)	385 (99%)		222 (97.8%)	269 (96.4%)	491 (97%)		
Yes	1 (0.5%)	3 (1.6%)	4 (1%)		5 (2.2%)	10 (3.6%)	15 (3%)		
**History of Various tumors**				0.688				0.214	
No	201 (98%)	182 (98.9%)	383 (98.5%)		219 (96.5%)	275 (98.6%)	494 (97.6%)		
Yes	4 (2%)	2 (1.1%)	6 (1.5%)		8 (3.5%)	4 (1.4%)	12 (2.4%)		
**History of Cerebrovascular disease**				0.509				0.231	
No	199 (97.1%)	181 (98.4%)	380 (97.7%)		226 (99.6%)	274 (98.2%)	500 (98.8%)		
Yes	6 (2.9%)	3 (1.6%)	9 (2.3%)		1 (0.4%)	5 (1.8%)	6 (1.2%)		
**History of Head trauma**				1				NA	
No	203 (99%)	183 (99.5%)	386 (99.2%)		227 (100%)	279 (100%)	506 (100%)		
Yes	2 (1%)	1 (0.5%)	3 (0.8%)		0 (0%)	0 (0%)	0 (0%)		
**History of Urinary tract infection**				0.223				1	
No	205 (100%)	182 (98.9%)	387 (99.5%)		225 (99.1%)	277 (99.3%)	502 (99.2%)		
Yes	0 (0%)	2 (1.1%)	2 (0.5%)		2 (0.9%)	2 (0.7%)	4 (0.8%)		
**History of Gout**				0.667				0.008**	
No	198 (96.6%)	180 (97.8%)	378 (97.2%)		209 (92.1%)	234 (83.9%)	443 (87.5%)		
Yes	7 (3.4%)	4 (2.2%)	11 (2.8%)		18 (7.9%)	45 (16.1%)	63 (12.5%)		
**History of Degenerative arthritis**				0.972				< 0.001***	
No	196 (95.6%)	177 (96.2%)	373 (95.9%)		202 (89%)	207 (74.2%)	409 (80.8%)		
Yes	9 (4.4%)	7 (3.8%)	16 (4.1%)		25 (11%)	72 (25.8%)	97 (19.2%)		
**History of Rheumatoid arthritis**				1				0.717	
No	203 (99%)	183 (99.5%)	386 (99.2%)		207 (91.2%)	258 (92.5%)	465 (91.9%)		
Yes	2 (1%)	1 (0.5%)	3 (0.8%)		20 (8.8%)	21 (7.5%)	41 (8.1%)		
**Steady state**				0.925				0.998	
Never	62 (30.2%)	55 (29.9%)	117 (30.1%)		64 (28.2%)	68 (24.4%)	132 (26.1%)		
< 30′	24 (11.7%)	22 (12%)	46 (11.8%)		39 (17.2%)	48 (17.2%)	87 (17.2%)		
30′~60′	43 (21%)	36 (19.6%)	79 (20.3%)		39 (17.2%)	51 (18.3%)	90 (17.8%)		
60′~90′	18 (8.8%)	21 (11.4%)	39 (10%)		27 (11.9%)	30 (10.8%)	57 (11.3%)		
90′~120′	21 (10.2%)	12 (6.5%)	33 (8.5%)		14 (6.2%)	20 (7.2%)	34 (6.7%)		
120′~180′	15 (7.3%)	18 (9.8%)	33 (8.5%)		24 (10.6%)	25 (9%)	49 (9.7%)		
180′~240′	10 (4.9%)	10 (5.4%)	20 (5.1%)		10 (4.4%)	16 (5.7%)	26 (5.1%)		
240′~300′	5 (2.4%)	4 (2.2%)	9 (2.3%)		2 (0.9%)	8 (2.9%)	10 (2%)		
> 300′	7 (3.4%)	6 (3.3%)	13 (3.3%)		8 (3.5%)	13 (4.7%)	21 (4.2%)		
**Sedentary life**				0.204				0.089	
Never	17 (8.3%)	9 (4.9%)	26 (6.7%)		16 (7%)	10 (3.6%)	26 (5.1%)		
< 30′	12 (5.9%)	11 (6%)	23 (5.9%)		10 (4.4%)	20 (7.2%)	30 (5.9%)		
30′~60′	12 (5.9%)	11 (6%)	23 (5.9%)		19 (8.4%)	33 (11.8%)	52 (10.3%)		
60′~90′	24 (11.7%)	10 (5.4%)	34 (8.7%)		23 (10.1%)	32 (11.5%)	55 (10.9%)		
90′~120′	17 (8.3%)	14 (7.6%)	31 (8%)		26 (11.5%)	17 (6.1%)	43 (8.5%)		
120′~180′	34 (16.6%)	24 (13%)	58 (14.9%)		35 (15.4%)	33 (11.8%)	68 (13.4%)		
180′~240′	21 (10.2%)	25 (13.6%)	46 (11.8%)		30 (13.2%)	48 (17.2%)	78 (15.4%)		
240′~300′	11 (5.4%)	10 (5.4%)	21 (5.4%)		21 (9.3%)	21 (7.5%)	42 (8.3%)		
> 300′	57 (27.8%)	70 (38%)	127 (32.6%)		47 (20.7%)	65 (23.3%)	112 (22.1%)		
**Mild exercise**				0.491				0.789	
Never	23 (11.2%)	19 (10.3%)	42 (10.8%)		5 (2.2%)	3 (1.1%)	8 (1.6%)		
< 30′	24 (11.7%)	26 (14.1%)	50 (12.9%)		11 (4.8%)	20 (7.2%)	31 (6.1%)		
30′~60′	30 (14.6%)	24 (13%)	54 (13.9%)		30 (13.2%)	50 (17.9%)	80 (15.8%)		
60′~90′	18 (8.8%)	25 (13.6%)	43 (11.1%)		34 (15%)	53 (19%)	87 (17.2%)		
90′~120′	31 (15.1%)	22 (12%)	53 (13.6%)		30 (13.2%)	28 (10%)	58 (11.5%)		
120′~180′	19 (9.3%)	16 (8.7%)	35 (9%)		36 (15.9%)	30 (10.8%)	66 (13%)		
180′~240′	13 (6.3%)	20 (10.9%)	33 (8.5%)		26 (11.5%)	38 (13.6%)	64 (12.6%)		
240′~300′	9 (4.4%)	5 (2.7%)	14 (3.6%)		10 (4.4%)	16 (5.7%)	26 (5.1%)		
> 300′	38 (18.5%)	27 (14.7%)	65 (16.7%)		45 (19.8%)	41 (14.7%)	86 (17%)		
**Moderate exercise**				0.986				0.996	
Never	92 (44.9%)	88 (47.8%)	180 (46.3%)		104 (45.8%)	146 (52.3%)	250 (49.4%)		
< 30′	21 (10.2%)	24 (13%)	45 (11.6%)		39 (17.2%)	35 (12.5%)	74 (14.6%)		
30′~60′	21 (10.2%)	21 (11.4%)	42 (10.8%)		29 (12.8%)	39 (14%)	68 (13.4%)		
60′~90′	17 (8.3%)	18 (9.8%)	35 (9%)		23 (10.1%)	29 (10.4%)	52 (10.3%)		
90′~120′	14 (6.8%)	12 (6.5%)	26 (6.7%)		10 (4.4%)	8 (2.9%)	18 (3.6%)		
120′~180′	12 (5.9%)	7 (3.8%)	19 (4.9%)		9 (4%)	10 (3.6%)	19 (3.8%)		
180′~240′	7 (3.4%)	5 (2.7%)	12 (3.1%)		3 (1.3%)	4 (1.4%)	7 (1.4%)		
240′~300′	6 (2.9%)	4 (2.2%)	10 (2.6%)		3 (1.3%)	2 (0.7%)	5 (1%)		
> 300′	15 (7.3%)	5 (2.7%)	20 (5.1%)		7 (3.1%)	6 (2.2%)	13 (2.6%)		
**Intense exercise**				0.088				0.956	
Never	91 (44.4%)	120 (65.2%)	211 (54.2%)		150 (66.1%)	193 (69.2%)	343 (67.8%)		
< 30′	12 (5.9%)	8 (4.3%)	20 (5.1%)		8 (3.5%)	10 (3.6%)	18 (3.6%)		
30′~60′	8 (3.9%)	3 (1.6%)	11 (2.8%)		8 (3.5%)	9 (3.2%)	17 (3.4%)		
60′~90′	6 (2.9%)	4 (2.2%)	10 (2.6%)		10 (4.4%)	11 (3.9%)	21 (4.2%)		
90′~120′	13 (6.3%)	2 (1.1%)	15 (3.9%)		5 (2.2%)	3 (1.1%)	8 (1.6%)		
120′~180′	10 (4.9%)	9 (4.9%)	19 (4.9%)		6 (2.6%)	8 (2.9%)	14 (2.8%)		
180′~240′	6 (2.9%)	6 (3.3%)	12 (3.1%)		3 (1.3%)	4 (1.4%)	7 (1.4%)		
240′~300′	7 (3.4%)	6 (3.3%)	13 (3.3%)		4 (1.8%)	8 (2.9%)	12 (2.4%)		
> 300′	52 (25.4%)	26 (14.1%)	78 (20.1%)		33 (14.5%)	33 (11.8%)	66 (13%)		

### Epigenome-wide association analysis of muscle status and gene from annotation results

**Fig. 1:**
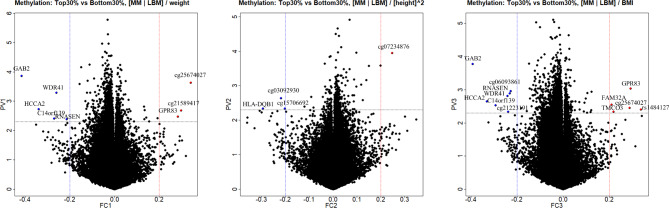
Volcano plot for men. **(A)** Weight adjustment group, **(B)** Height^2^ adjustment group, **(C)** BMI adjustment group

**Fig. 2:**
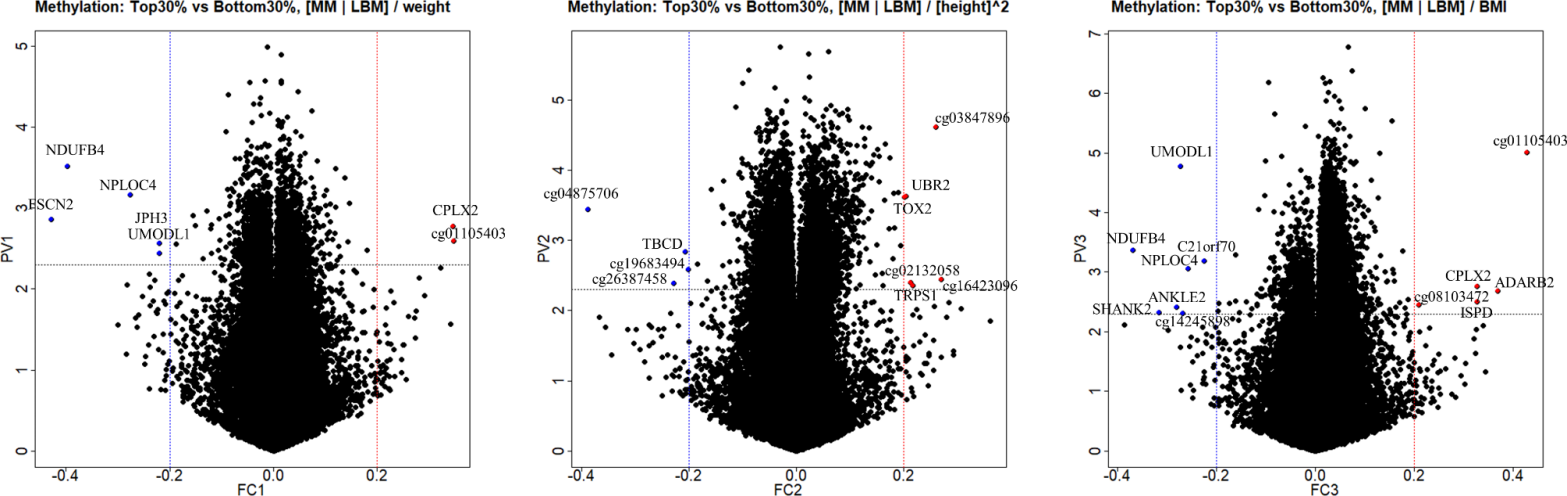
Volcano plot for women. **(A)** Weight adjustment group, **(B)** Height^2^ adjustment group, **(C)** BMI adjustment group

Among 446 participants, 44 met the criteria and were included in T30 or B30 group. Finally, the analysis included these 44 subjects whose DNA methylation profiles were investigated. Based on epigenome-wide association analysis of DNA methylation, genes from annotation results of each group were certified using a volcano plot (Figs. 1 and 2). In men group through MMI1 method, five down-regulated genes (GAB2, WDR41, HCCA2, C14orf139, and RNASEN) and one up-regulated gene (GPR83) were associated with middle-age muscle loss. In women group through MMI1 method (|Log2 fold change| > 0.21 and P < 0.05), five down-regulated genes (JPH3, FSCN2, UMODL1, NPLOC4, and NDUFB4) and one up-regulated gene (CPLX2) were associated with middle-age muscle loss. In men group through MMI2 method, there was only one down-regulated gene (HLA-DQB1). However, in women group through MMI2 method, there were one down-regulated gene (TBCD) and three up-regulated genes (TRPS1, UBR2, TOX2). In MMI3 method, several muscle-loss-related genes were found in men and women: four down-regulated genes (C14orf139, HCCA2, RNASEN, GAB2) and three up-regulated genes (FAM32A, TMCO3, GPR83) in men; seven down-regulated genes (WDR41, ANKLE2, UMODL1, SHANK2, C21orf70, NPLOC4, NDUFB4) and three up-regulated genes (CPLX2, ISPD, ADARB2) in women group. All results of muscle atrophy-related genes are shown in Table 4.

**Table 4:** CpG sites that show significant differences in DNA methylation level and CpG sites related to DEGs.

MMI 1															
**Man**	**CpG Site**	**AccNo**	**Chr**	**Location**	**Gene from annotation results**	**Diffexpressed**	**FC**	**logPV**	**Sig.**						
	cg24769381	NM_080491	chr11	78,052,863	GAB2	DOWN	-0.415643811892857	3.86328542675402	**						
	cg25420101	NM_018268	chr5	76,785,615	WDR41	DOWN	-0.260063782714286	3.29157387971701	*						
	cg14089103	NM_053005	chr11	1,682,006	HCCA2	DOWN	-0.339951531642857	2.73357398106781	*						
	cg19680693	NM_016540	chr11	94,111,807	GPR83	UP	0.283395011053571	2.47597290720169	*						
	cg10006614	NR_026779	chr14	95,877,336	C14orf139	DOWN	-0.26958618975	2.41246956207361	*						
	cg23230564	NM_013235	chr5	31,470,890	RNASEN	DOWN	-0.213472371535714	2.39352138883114	*						
	cg25674027	-	chr12	103,325,781	-	UP	0.340683558	3.636438473	**						
	cg21589417	-	chr2	170,834,853	-	UP	0.2970822	2.687368	*						
**Woman**	**CpG Site**	**AccNo**	**Chr**	**Location**	**Gene from** **annotation results**	**Diffexpressed**	**FC**	**logPV**							
	cg02157463	NM_020655	chr16	87,648,182	JPH3	DOWN	-0.219644786428571	2.5666288885608	*						
	cg05248234	NM_001077182	chr17	79,495,519	FSCN2	DOWN	-0.4283696875	2.85956402441411	*						
	cg08880261	NM_173568	chr21	43,547,872	UMODL1	DOWN	-0.220094758928571	2.44280687166786	*						
	cg11128983	NM_001008220	chr5	175,297,531	CPLX2	UP	0.345062625	2.77022881427118	*						
	cg16835531	NM_017921	chr17	79,524,882	NPLOC4	DOWN	-0.276650375	3.16122080151316	*						
	cg23737713	NM_004547	chr3	120,318,272	NDUFB4	DOWN	-0.397565735714286	3.5131539957498	**						
	cg01105403	-	chr2	240,723,304	-	UP	0.346911877	2.591143579	*						
**MMI 2**															
**Man**	**CpG Site**	**AccNo**	**Chr**	**Location**	**Gene from** **annotation results**	**Diffexpressed**	**FC**	**logPV**							
	cg19301366	NM_002123	chr6	32,627,845	HLA-DQB1	DOWN	-0.293714560909091	2.34324806451094	*						
	cg07234876	-	chr8	600,039	-	UP	0.248053686	3.953221928	**						
	cg15706692	-	chr6	75,775,380	-	DOWN	-0.202074557	2.338389888	*						
	cg03092930	-	chr2	132,586,330	-	DOWN	-0.2180954	2.643175	*						
**Woman**	**CpG Site**	**AccNo**	**Chr**	**Location**	**Gene from** **annotation results**	**Diffexpressed**	**FC**	**logPV**							
	cg02398342	NM_005993	chr17	80,708,632	TBCD	DOWN	-0.206867782142857	2.84185096284346	*						
	cg04613734	NM_014112	chr8	116,663,921	TRPS1	UP	0.216482961071429	2.35037125914594	*						
	cg20646500	NM_015255	chr6	42,536,105	UBR2	UP	0.203217782142857	3.62275825563645	**						
	cg20889774	NM_001098796	chr20	42,548,586	TOX2	UP	0.200653505357143	3.61121530175873	**						
	cg02132058	-	chr3	170,451,961	-	Up	0.2122729	2.397384868	*						
	cg03847896	-	chr1	112,154,295	-	Up	0.25967692	4.607922587	***						
	cg04875706	-	chr15	97,311,928	-	Down	-0.387855923	3.443149465	**						
	cg16423096	-	chr14	22,279,816	-	Up	0.269297214	2.441342191	*						
	cg19683494	-	chr5	74,908,142	-	Down	-0.201116741	2.58145445	*						
	cg26387458	-	chr11	28,642,652	-	Down	-0.228649507	2.384148658	*						
**MMI 3**															
**Man**	**CpG Site**	**AccNo**	**Chr**	**Location**	**Gene from** **annotation results**	**Diffexpressed**	**FC**	**logPV**							
	cg07848485	NM_014077	chr19	16,302,087	FAM32A	UP	0.210522475504273	2.55418583600442	*						
	cg07876831	NM_017905	chr13	114,161,463	TMCO3	UP	0.218773657307692	2.33447658735994	*						
	cg10006614	NR_026779	chr14	95,877,336	C14orf139	DOWN	-0.294184812299145	2.53237921014287	*						
	cg14089103	NM_053005	chr11	1,682,006	HCCA2	DOWN	-0.332116075222222	2.64859352814776	*						
	cg19680693	NM_016540	chr11	94,111,807	GPR83	UP	0.292507552188034	3.03538507339969	*						
	cg23230564	NM_013235	chr5	31,470,890	RNASEN	DOWN	-0.232108471931624	2.89123940510241	*						
	cg24769381	NM_080491	chr11	78,052,863	GAB2	DOWN	-0.393460424897436	3.77686383392583	**						
	cg06093861	-	chr7	75,780,412	-	DOWN	-0.228174596	2.959060269	*						
	cg21223191	-	chr6	32,583,741	-	DOWN	-0.238787895	2.339663008	*						
	cg25420101	NM_018268	chr5	76,785,615	WDR41	DOWN	-0.242817133	2.809440947	*						
	cg25674027	-	chr12	103,325,781	-	UP	0.288913633	2.452334626	*						
	rs1484127	-	chr8	51,888,207	-	UP	0.3369222	2.403778	*						
**Woman**	**CpG Site**	**AccNo**	**Chr**	**Location**	**Gene from** **annotation results**	**Diffexpressed**	**FC**	**logPV**							
	cg07249488	NM_015114	chr12	133,304,604	ANKLE2	DOWN	-0.281426491428571	2.4185295012601	*						
	cg08880261	NM_173568	chr21	43,547,872	UMODL1	DOWN	-0.273448150476191	4.7760813124593	***						
	cg09157251	NM_012309	chr11	70,733,251	SHANK2	DOWN	-0.315829999047619	2.3229259734005	*						
	cg11128983	NM_001008220	chr5	175,297,531	CPLX2	UP	0.32623238952381	2.76421772471434	*						
	cg11401796	NM_058190	chr21	46,378,438	C21orf70	DOWN	-0.225213455238095	3.18526913733318	*						
	cg11973981	NM_001101426	chr7	16,457,583	ISPD	UP	0.326718622857143	2.49869379173893	*						
	cg16835531	NM_017921	chr17	79,524,882	NPLOC4	DOWN	-0.257372975714286	3.05248496664675	*						
	cg20205188	NM_018702	chr10	1,251,771	ADARB2	UP	0.369248260380952	2.68913280913789	*						
	cg23737713	NM_004547	chr3	120,318,272	NDUFB4	DOWN	-0.369890199047619	3.37327384575536	**						
	cg01105403	-	chr2	240,723,304	-	UP	0.427513028	5.010325703	***						
	cg08103472	-	chr15	40,731,325	-	UP	0.209570254	2.457436576	*						
	cg14245898	-	chr19	15,248,535	-	DOWN	-0.268531019	2.307447725	*						

**logPV: -log**_**10**_(p-value), *p < 5*10^-3, **p < 5*10^-4, ***p < 5*10^-5

### Enrichment analysis of DMRs

**Fig. 3:**
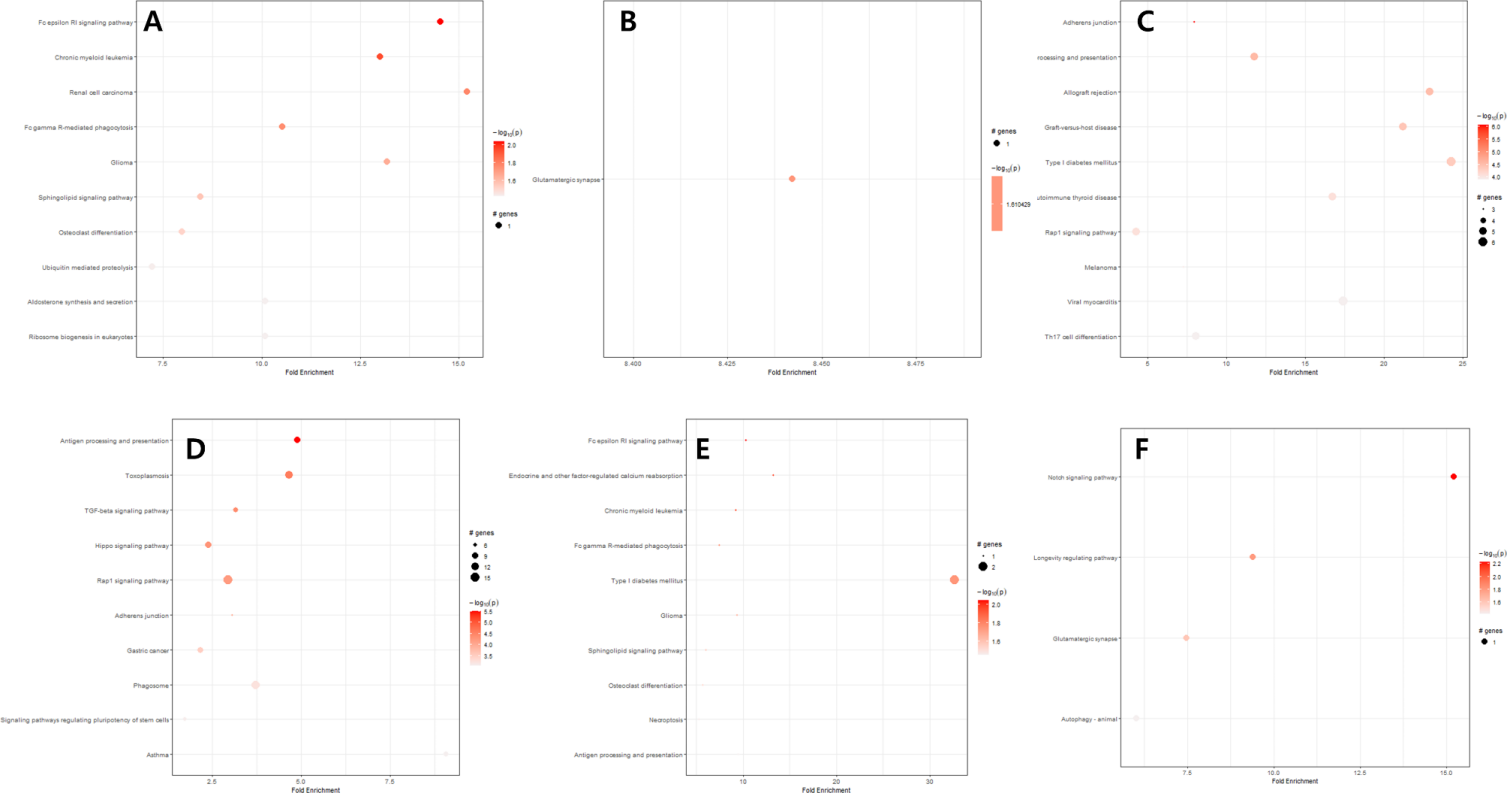
Enrichment analysis of DMRs in each group. **(A)** Men with weight adjustment, **(B)** Women with weight adjustment, **(C)** Men with square of height adjustment, **(D)** Women with square of height adjustment, **(E)** Men with BMI adjustment, **(F)** Women with BMI adjustment

Enrichment analysis of DMRs are presented in Fig. 3. In men group through MMI1 method (|Log2 fold change| > 0.21 and P < 0.05), Fc epsilon RI signaling pathway which is associated with inducing mast cell degranulation and plays a critical role in human airway smooth muscle cell function was the most enriched with statistical significance [23]. In women group through MMI1 method (|Log2 fold change| > 0.21 and P < 0.05), only the glutamatergic synapse pathway showed a significant result. Glutamate is known to act as a mediator between the motor nerve tip and skeletal muscle fibers [24]. In men group through MMI2 method (|Log2 fold change| > 0.07 and P < 0.05), the adherens junction pathway that join mature myocytes and give myofibrils places to attach to the membrane [25]was the most significant. Moreover, the Rap1 signaling pathway associated with skeletal muscle cell differentiation also showed significant results in the same group [26]. Women group through MMI2 method (|Log2 fold change| > 0.07 and P < 0.05), showed similar results, having an enriched Rap1 signaling pathway. In men group through MMI3 method (|Log2 fold change| > 0.21 and P < 0.05), the Fc epsilon RI signaling pathway was the most enriched. Particularly, the notch signaling pathway considered as a key player in skeletal muscle regeneration and development [27] was significantly enriched in women group through MMI3 method (|Log2 fold change| > 0.21 and P < 0.05). According to these results, changes in pathways of genes related to DMR between T30 and B30 might be due to differences in adjustment method rather than due to gender differences.

## Discussion

The present epigenetic nationwide cohort study was conducted based on MMI adjusted by weight, square of height, and BMI. In the group of men adjusted by weight, there were significant differences in BMI, the circumference of waist, and the circumference of hip. These obesity-related features are associated with muscle loss [28]. Smoking also showed a significant difference in the present study. One study has reported that smoking is associated with muscle loss in men [29]. Sedentary life known to be associated with sarcopenia or muscle loss was also significantly different in the present study. In a group of women adjusted by weight, MMI values were decreased with increasing age [30]. The relationship between age and muscle loss showed relevances in many studies [31–34].In addition, there were significant differences in people with a history of high blood pressure and diabetes, both of which are known to be associated with sarcopenia or muscle loss [35–38].

Through the MMI2 method, age, BMI, waist, and hip revealed significant results in the men group. Significant results were also observed in individuals with a history of asthma illness. The decline in respiratory muscle mass, function, and power could be related to aging [39]. Several studies have consistently revealed the association between respiratory diseases such as asthma and sarcopenia or muscle loss [39, 40]. In addition, the men group through MMI2 method showed a significant difference in sedentary life. In the women group through MMI2 method, there were significant differences in BMI, the circumference of waist, and hip. However, there was no significant result according to age difference. In particular, there was a significant difference in people with a history of degenerative arthritis. As muscle wasting is a natural part of aging, sarcopenia prevalence has been recently proven in individuals with OA [41, 42]. It has been suggested that muscle wasting has a direct impact on joint stability and that loss of mobility can lead to articular cartilage degeneration [43].

In the group of men adjusted by BMI, there were significant differences in BMI, the circumference of waist and hip, and smoke. However, there was no significant difference in sedentary life. In the group of men adjusted by BMI, there were significant differences in age, BMI, waist, hip, history of hypertension, history of diabetes, and history of degenerative arthritis. Additionally, people with a history of gout showed low adjusted MMI results. According to a study by KM Beavers, reduced skeletal muscle mass is linked to increased serum uric acid levels [44]. Many studies have shown that gout might be related to muscle mass loss or sarcopenia [45, 46].

DNA methylation may play an important role in muscle loss during aging [20]. Therefore, finding the characteristics of age-related DNA methylation change might be the most promising way to find biomarkers of muscle aging [47, 48]. Several studies have determined methylation levels of certain representative genes in young and old people and found an age-related increase in methylation between NDUFB6 and COX7A1 that is important in metabolic mechanisms [49, 50].

Likewise, a gene annotation study using DMR was performed in this study. In the group of men adjusted by weight, GAB2 was a downregulated gene. According to a study by Eric Edstrom et al., Shc-GRB2-GAB (Shc, Src homology 2 domain-containing; GRB2, growth factor receptor bound protein-2; GAB, GRB2 associated binding protein) adaptor might interact with IGF-1 receptor, which activates PI3K-AKT. Through this pathway, AKT could block atrophy and stimulate myofiber hypertrophy [51]. In contrast, JPH3 was a downregulated gene in the group of women adjusted by weight. Junctophilins are major components responsible for the synthesis of JMCs (junctional membrane complexes) in skeletal and cardiac muscles [52]. Li et al. have suggested that JPH3 plays a critical function in maintaining the proper distance and crosstalk between the ER and mitochondria via the Pgc-1 pathway in beta cells [53]. Pgc-1α in nuclei is known to promote target molecule transcription in skeletal muscles [53]. Muscle atrophy-related genes in the weight adjustment group is summarized in Supplementary Table 1.

In the group of men adjusted by height squared, HLA-DQB1 was a downregulated gene. According to a study by Singh et al., HLA-DQB1 is specifically enriched in skeletal muscles and highly associated with the handgrip trait [54]. HLA-DQB1 is nominally associated with sarcopenia (EWGSOP combined definition). The influence of HLA type is more significant in women than in men [55, 56]. In the group of women adjusted by height square, TBCD was a downregulated gene and UBR2 was an upregulated gene. Carrio et al. have suggested that the TBCD gene is one gene with differentially methylated CpG (dmCpG) affected by aging. The most enriched words and pathways among genes with two or more intragenic dmCpG sites were “muscle cell” (*P* = 0.0004), indicating that dmCpG sites found in the elderly were related to muscle tissue functions and neuromuscular junctions [57]. The TBCD gene has the most intragenic dmCpG sites (46 distinct sites, or 13.2% of the total number of CpG sites in the gene) [58]. Additionally, Ubr2 is up-regulated in disuse atrophying skeletal muscles of mice [59]. Muscle atrophy-related genes in the square of height adjustment group are summarized in Supplementary Table 2.

**Fig. 4:**
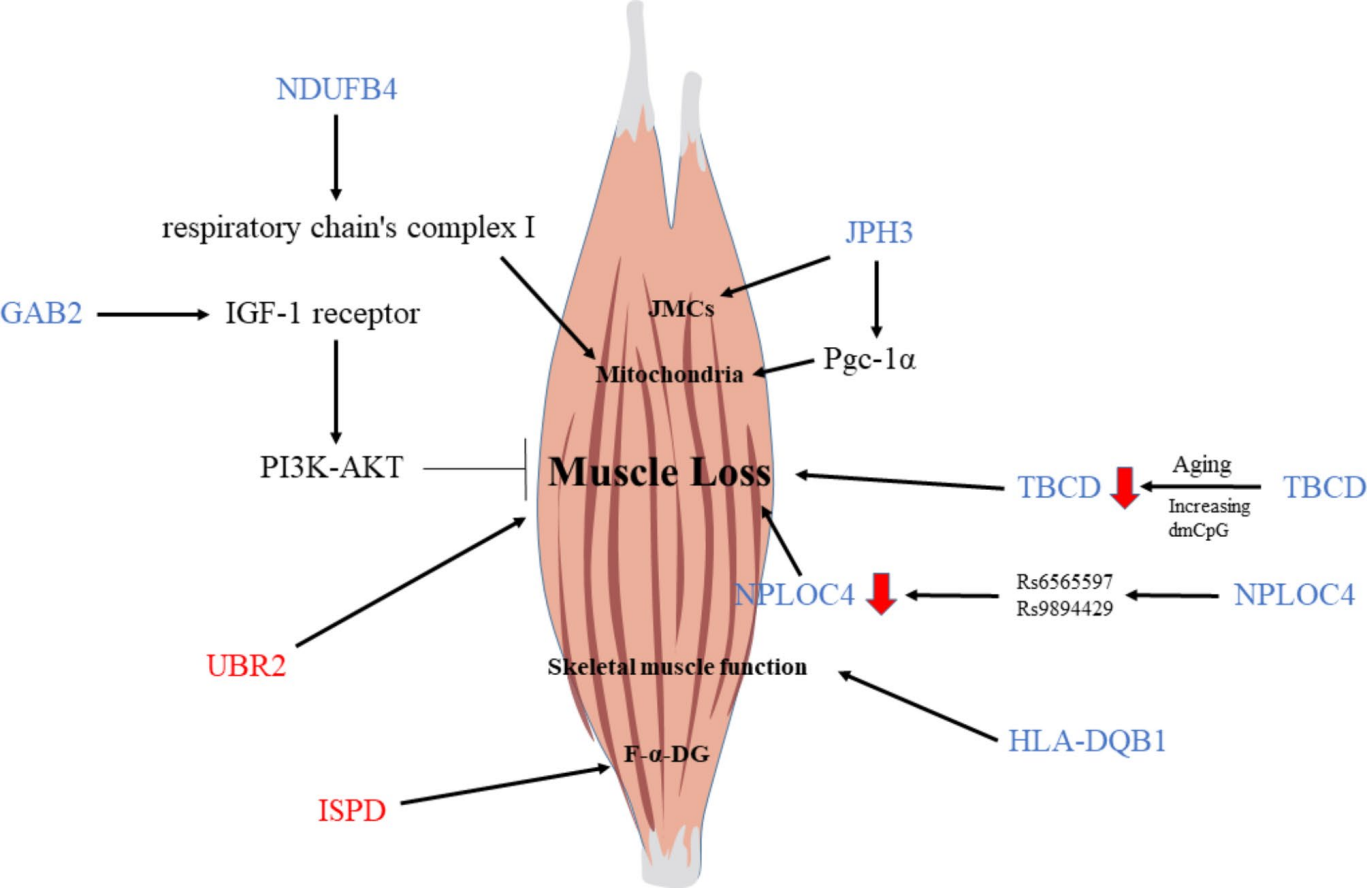
Overall molecular mechanisms related to muscle loss. Blue letters mean down – regulated genes and red letters mean up-regulated genes

The group of men adjusted by BMI also showed GAB2 as a downregulated gene. On the other hand, the group of women adjusted by BMI showed two downregulated genes (NPLOC4 and NDUFB4) and one upregulated gene (ISPD) known to be associated with muscle loss. NPLOC4 is known as a muscle degeneration-related gene because it has two SNPs (rs6565597 and rs9894429) that are related to age-related macular degeneration [60, 61]. NDUFB4 is also shown to be higher in muscles. It codes for subunits of the respiratory chain’s complex I which is related to energy metabolism.^56^ However, ISPD was upregulated in the low-adjusted MMI group. Marcela P. Cataldi has shown that ISPD overexpression increases functional glycosylation of α subunit of dystroglycan (F-α-DG) in skeletal muscles [62]. Because it is known that dystroglycan-null could be caused by muscle dystrophy, the muscle loss group may compensatively show ISPD upregulation. Muscle atrophy-related genes in the weight adjustment group are summarized in Supplementary Table 3. Overall molecular mechanisms related to muscle loss are presented in Fig. 4.

In conclusion, this study presents results about which factor should be concerned first in MMI adjustment. It could enable future epigenetic studies of genes based on annotation results. The present study is a nationwide study in Korea with the largest size up to date that compares adjustment methods for MMI in epigenetic research.

There are some limitations in the present study. First, compared to a large number of participants, the number of genetic tests was insufficient. In addition, because it was a cohort study, there was no restriction on the composition of participants. Tests for sarcopenia screening such as the handgrip test were not conducted. Participants were a middle-age group. There were no results from elderly participants. Second, eQTL analysis was not performed. If eQTL analysis could be added later in an extended cohort study, it will be an in-depth study on the difference between genotype and phenotype of sarcopenia. Therefore, further study including elderly participants with sufficient genetic tests is needed.

### Electronic supplementary material

Below is the link to the electronic supplementary material.

Supplementary Material 1

## Data Availability

The data presented in the study are included in the article. Further inquiries can be directed to the corresponding authors.
