# Supplementary material for: Relationship between DNA methylation changes and skeletal muscle mass
Source: BMC Genom Data. 2023 Aug 31;24:48. doi: 10.1186/s12863-023-01152-3 (PMC10472633; doi:10.1186/s12863-023-01152-3)
Supplement: Supplementary file 1 — Supplementary Material 1 [file 12863_2023_1152_MOESM1_ESM.docx]

**Supplementary Table 1. Summary of muscle atrophy related genes in the weight adjustment group**

| **Men** | | | |
| --- | --- | --- | --- |
| **Gene name** | **Associated studies** | | **Summary of study** |
| **Down** | | | |
| GAB2 | Edström et al., 2006 | | Shc-GRB2-GAB (Shc, Src homology 2 domain containing; GRB2, growth factor receptor bound protein-2; GAB, GRB2 associated binding protein) adaptor might interact with IGF-1 receptor, which activate PI3K-AKT. Through this pathway, AKT would block atrophy and stimulate myofiber hypertrophy by its pathway. |
| **Women** | | | |
| **Gene name** | | **Subject of article** | **Summary of study** |
| **Down** | | | |
| JPH3 | | Piggott and Jin, 2021  Li et al., 2016 | Junctophilins are the major component responsible for the synthesis of JMCs (junctional membrane complexes) in skeletal and cardiac muscles, according to extensive research.  In beta cells, JPH3 plays a critical function in maintaining the proper distance and crosstalk between the ER and mitochondria via the Pgc-1 pathway. Pgc-1α in nuclei is known as promoting the target molecules transcription in skeletal muscle. |

**Supplementary Table 2. Summary of muscle atrophy related genes in the square of height adjustment group**

| **Men** | | |
| --- | --- | --- |
| **Gene name** | **Subject of article** | **Summary of study** |
| **Down** | | |
| HLA-DQB1 | Jones et al., 2020, 2021  Singh and Gasman, 2021 | HLA-DQB1 was nominally associated with sarcopenia (EWGSOP combined definition) and influence of HLA types is more significant in women.  HLA-DQB1 is specifically enriched in skeletal muscle and highly associated with hand grip trait. |
| **Women** | | |
| **Gene name** | **Subject of article** | **Summary of study** |
| **Down** | | |
| TBCD | Carrió and Suelves, 2015  Zykovich et al., 2014 | One example of a gene with dmCpG that is affected by aging is the tubulin-folding cofactor D (TBCD) gene. The most enriched words and pathways among genes with two or more intragenic dmCpG sites were "muscle cell" (P = 0.0004), indicating that the dmCpG sites found in the elderly were related to muscle tissue functions and neuromuscular junctions.  The TBCD gene has the most intragenic dmCpG sites (46 distinct sites, or 13.2 percent of the total number of CpG sites in the gene). |
| **UP** | | |
| UBR2 | Hockerman et al., 2014 | Ubr2 was up-regulated in disuse atrophying skeletal muscle of mice. |

**Supplementary Table 3. Summary of muscle atrophy related genes in the BMI adjustment group**

| **Men** | | |
| --- | --- | --- |
| **Gene name** | **Subject of article** | **Summary of study** |
| **Down** | | |
| GAB2 | Edström et al., 2006 | Shc-GRB2-GAB (Shc, Src homology 2 domain containing; GRB2, growth factor receptor bound protein-2; GAB, GRB2 associated binding protein) adapter might interact with IGF-1 receptor, which activates PI3K-AKT. Through this pathway, AKT would block atrophy and stimulate myofiber hypertrophy by its pathway. |
| **Women** | | |
| **Gene name** | **Subject of article** | **Summary of study** |
| **Down** | | |
| NPLOC4 | Fritsche et al., 2016  Liu et al., 2010 | NPLOC4 has two SNPs (rs6565597 and rs9894429) that are linked to age-related macular degeneration. |
| NDUFB4 | Kobilo et al., 2014 | NDUFB4 expression, which codes for subunits of the respiratory chain's complex I, was shown to be higher in muscles, which is related to energy metabolism. |
| **Up** | | |
| ISPD | Cataldi et al., 2019 | ISPD overexpression increase F-α-DG (functional glycosylation of α subunit of dystroglycan) in skeletal muscle. |

Carrió, E., and Suelves, M. (2015). DNA methylation dynamics in muscle development and disease. *Front Aging Neurosci* 7, 19. doi: 10.3389/fnagi.2015.00019.

Cataldi, M. P., Blaeser, A., Lu, P., Leroy, V., and Lu, Q. L. (2019). ISPD Overexpression Enhances Ribitol-Induced Glycosylation of α-Dystroglycan in Dystrophic FKRP Mutant Mice. *Mol Ther Methods Clin Dev* 17, 271–280. doi: 10.1016/j.omtm.2019.12.005.

Edström, E., Altun, M., Hägglund, M., and Ulfhake, B. (2006). Atrogin-1/MAFbx and MuRF1 are downregulated in aging-related loss of skeletal muscle. *J Gerontol A Biol Sci Med Sci* 61, 663–674. doi: 10.1093/gerona/61.7.663.

Fritsche, L. G., Igl, W., Bailey, J. N. C., Grassmann, F., Sengupta, S., Bragg-Gresham, J. L., et al. (2016). A large genome-wide association study of age-related macular degeneration highlights contributions of rare and common variants. *Nat Genet* 48, 134–143. doi: 10.1038/ng.3448.

Hockerman, G. H., Dethrow, N. M., Hameed, S., Doran, M., Jaeger, C., Wang, W.-H., et al. (2014). The Ubr2 Gene is Expressed in Skeletal Muscle Atrophying as a Result of Hind Limb Suspension, but not Merg1a Expression Alone. *Eur J Transl Myol* 24, 3319. doi: 10.4081/ejtm.2014.3319.

Jones, G., Pilling, L. C., Kuo, C.-L., Kuchel, G., Ferrucci, L., and Melzer, D. (2020). Sarcopenia and Variation in the Human Leukocyte Antigen Complex. *J Gerontol A Biol Sci Med Sci* 75, 301–308. doi: 10.1093/gerona/glz042.

Jones, G., Trajanoska, K., Santanasto, A. J., Stringa, N., Kuo, C.-L., Atkins, J. L., et al. (2021). Genome-wide meta-analysis of muscle weakness identifies 15 susceptibility loci in older men and women. *Nat Commun* 12, 654. doi: 10.1038/s41467-021-20918-w.

Kobilo, T., Guerrieri, D., Zhang, Y., Collica, S. C., Becker, K. G., and van Praag, H. (2014). AMPK agonist AICAR improves cognition and motor coordination in young and aged mice. *Learn Mem* 21, 119–126. doi: 10.1101/lm.033332.113.

Li, L., Pan, Z.-F., Huang, X., Wu, B.-W., Li, T., Kang, M.-X., et al. (2016). Junctophilin 3 expresses in pancreatic beta cells and is required for glucose-stimulated insulin secretion. *Cell Death Dis* 7, e2275. doi: 10.1038/cddis.2016.179.

Liu, F., Wollstein, A., Hysi, P. G., Ankra-Badu, G. A., Spector, T. D., Park, D., et al. (2010). Digital Quantification of Human Eye Color Highlights Genetic Association of Three New Loci. *PLOS Genetics* 6, e1000934. doi: 10.1371/journal.pgen.1000934.

Piggott, C. A., and Jin, Y. (2021). Junctophilins: Key Membrane Tethers in Muscles and Neurons. *Frontiers in Molecular Neuroscience* 14, 141. doi: 10.3389/fnmol.2021.709390.

Singh, A. N., and Gasman, B. (2021). Disentangling the Genetics of Sarcopenia: prioritization of NUDT3 and KLF5 as genes for lean mass and HLA-DQB1-AS1 for hand grip strength based on associated SNPs. doi: 10.21203/rs.2.16139/v2.

Zykovich, A., Hubbard, A., Flynn, J. M., Tarnopolsky, M., Fraga, M. F., Kerksick, C., et al. (2014). Genome-wide DNA methylation changes with age in disease-free human skeletal muscle. *Aging Cell* 13, 360–366. doi: 10.1111/acel.12180.
